# Minibactenecins ChBac7.Nα and ChBac7. Nβ - Antimicrobial Peptides from Leukocytes of the Goat Capra hircus.

**Published:** 2016

**Authors:** O.V. Shamova, D.S. Orlov, M.S. Zharkova, S.V. Balandin, E.V. Yamschikova, D. Knappe, R. Hoffmann, V.N. Kokryakov, T.V. Ovchinnikova

**Affiliations:** Institute of Experimental Medicine, Acad. Pavlov Str. 12, Saint-Petersburg, 197376, Russia; M.M. Shemyakin and Yu.A. Ovchinnikov Institute of Bioorganic Chemistry of the Russian Academy of Sciences, Mikhluho-Maklaya Str. 16/10, Moscow, 117997, Russia; Saint-Petersburg State University, Universitetskaya Emb, 7/9, Saint-Petersburg, 199034, Russia; University of Leipzig, Deutscher Platz 5, D-04103 Leipzig, Germany

**Keywords:** antimicrobial peptides, cathelicidins, mini-bactenecins

## Abstract

Antimicrobial peptides (AMPs) of neutrophils play an important role in the animal and human host defenses. We have isolated two AMPs (average molecular masses of 2895.5 and 2739.3 Da), with potent antimicrobial activity from neutrophils of the domestic goat *(Capra hircus). *A structural analysis of the obtained peptides revealed that they encompass N-terminal fragments (1–21 and 1–22) of the proline-rich peptide bactenecin 7.5. The primary structure of caprine bactenecin 7.5 had been previously deduced from the nucleotide sequence, but the corresponding protein had not been isolated from leukocytes until now. The obtained caprine AMPs were designated as mini-batenecins (mini-ChBac7.5Nα and mini-ChBac7.5Nβ), analogously to the reported C-terminal fragment of the ovine bactenecin 7.5 named Bac7.5mini [Anderson, Yu, 2003]. Caprine mini-ChBac7.5Nα and mini-ChBac7.5Nβ exhibit significant antimicrobial activity against Gram-negative bacteria, including drug-resistant strains of *Pseudomonas aeruginosa*, *Klebsiella spp*., *Acinetobacter baumannii *at a range of concentrations of 0.5–4 μM, as well as against some species of Gram-positive bacteria (*Listeria monocytogenes *EGD, *Micrococcus luteus*). The peptides demonstrate lipopolysaccharide-binding activity. Similarly to most proline-rich AMPs, caprine peptides inactivate bacteria without appreciable damage of their membranes. Mini-ChBac7.5Nα and mini-ChBac7.5Nβ have no hemolytic effect on human red blood cells and are nontoxic to various cultured human cells. Therefore, they might be considered as promising templates for the development of novel antibiotic pharmaceuticals. Isolation of highly active fragments of the antimicrobial peptide from goat neutrophils supports the hypothesis that fragmentation of cathelicidin-related AMPs is an important process that results in the generation of potent effector molecules, which are in some cases more active than full-size AMPs. These truncated AMPs may play a crucial role in host defense reactions.

## INTRODUCTION

Antimicrobial peptides (AMPs) are cationic molecules contained in leukocytes, barrier epithelial cells, and other cell types, and they are involved in the protection of humans and animals against infectious agents. Along with antimicrobial action, AMPs have other properties, including immunomodulatory activity, which suggest that these compounds can be prototypes for new complex antibiotic drugs. From this perspective, cathelicidin- related AMPs, a large group of peptides widely present in vertebrates, are of particular interest. The peptides of this protein family are generated from precursor proteins by proteolytic cleavage of the N-terminal portion (cathelin-like domain) from the C-terminal region, corresponding to mature AMP. Proteolysis initiates upon activation of neutrophils and barrier epithelial cells during infectious processes. In some cathelicidins, for example human cathelicidin LL-37, mature AMP molecules are also subjected to processing [1], which leads to the formation of fragments with their own specific ranges of biological effects, including antibacterial, antitumor, and other types of activity. A similar proteolytic cleavage of peptides has been also described for ovine bactenecins [2]. It is assumed that the fragmentation of mature AMPs has a biological meaning and that these fragments may play a key role in multiple types of defense response [1, 2].

Among the currently known AMPs, cathelicidins of artiodactyl animas are of special interest due to their high antimicrobial activity and combination of properties, which make these peptides promising for practical application. The peptides isolated from the leukocytes of artiodactyls include the following AMPs: porcine protegrins, PR-39 [3, 4]; bovine bactenecins, BMAP-27 and BMAP-28, dodecapeptide, indolicidin [5-8]; ovine SMAP-29 [9], etc. Some of these peptides have been selected as targets for detailed research aimed at drug design. Interestingly, the neutrophils of some artiodactyls, including goats, contain no defensin-derived AMPs [10], suggesting the crucial role of cathelicidins in the protection of these animals against infections. Thus, the study of the neutrophilic AMPs of artiodactyl animals is important for both a potential discovery of new biologically active molecules, which can serve as templates for new drug design, and for the development of the fundamental concepts of cathelicidin’s role in host defense. The present work is aimed at discovering and characterizing new leukocytic AMPs of the domestic goat *Capra hircus. *Previously, we had isolated two peptides, bactenecins ChBac5 and ChBac3.4 [11, 12], from caprine leukocytes. In this paper, other *AMPs* have been studied*.*

## EXPERIMENTAL

**Reagents**

We used sodium chloride (S9625), tris-(hydroxymethyl) aminomethane (T1503), agarose (Type I, low EEO, A6013) trifluoroacetic (302031) and heptafluorobutyric (52411) acids, o-nitrophenyl-β-galactopyranoside (N1127), MTT (3-(4,5-dimethylthiazol-2-yl)-2,5-diphenyltetrazoliumbromide; M5655), cetyltrimethylammonium bromide (H6269), Sigma, USA; nitrocefin (484400), Calbiochem, USA; acetic acid, ammonium chloride, sodium acetate, Vekton, Russia; fetal calf serum (1.1.8.3.), RPMI-1640 (1.3.4) and DMEM (1.3.5.1.) culture media for cell cultures, Biolot, Russia; Sabouraud culture medium (broth), Research Center of Pharmacotherapy, Russia; Mueller Hinton nutrient broth (M391), HiMedia, India. Chemically synthesized peptides, protegrin 1 provided courtesy of R. Lehrer (University of California, Los Angeles, USA) and bactenecins ChBac5, ChBac5 20-43 and ChBac3.4 provided courtesy of N.I. Kolodkin (State Research Institute of Pure Biochemicals of the Federal Medical and Biological Agency), were used as reference peptides.

**Isolation and purification of antimicrobial peptides from leucocytes of the domestic goat**

A fraction of white cells enriched with neutrophils was obtained from blood of healthy adult goats (C. hircus). Erythrocyte hemolysis was carried out with an ammonium chloride solution. One liter of whole blood was processed to obtain 2.5 g of leukocytes (wet weight). We used two options of protein extraction. In the first case, the cells were destroyed by homogenization in a 10% acetic acid solution, and the homogenate was suspended with a magnetic stirrer at 4°C for 18–24 h, and then centrifuged at 15,000 *g *for 1 hour. The supernatant was dried and reconstituted in 0.1 M Tris-HCl-buffer, pH 7.5, and incubated at 37°C for 4 hours to digest the cathelicidin precursors. In the second case, the extraction was carried out using a 0.3% cetyltrimethylammonium bromide solution in 0.02 M sodium acetate buffer, pH 4.5. When using this extraction method, we created the conditions for enzymatic reactions as early as during the extraction process. The material resulted from the extraction was ultrafiltered through a YM-10 membrane (NMWCO of 10 kDa) from Amicon (USA) for separation of the low-molecular-weight protein fraction and further concentrated and desalted using ultrafiltration through the YM-1 membrane (NMWCO of 1 kDa). The material containing acid-soluble polypeptides with a molecular weight of less than 10–15,000 Da was placed in a column for electrophoretic separation using preparative continuous elution electrophoresis (CEE) in 12.5% polyacrylamide gel in the acidic buffer system with urea [13], using the Bio-Rad instrument (USA). The fractions with detected antimicrobial activity were collected, and the peptides in these fractions were separated by several consecutive cycles of reverse-phase high-performance liquid chromatography (RP-HPLC) on a Gold System instrument from Beckman (USA) using Vydac C-18 columns (4.6 × 250 mm; sorbent particle size of 5 μm). The purity of the fractions obtained after RP-HPLC was assessed by analytical electrophoresis [14], mass spectrometry, and analytical RP-HPLC. The protein concentration in the purified preparations was determined by the Bradford’s method and Wolf’s method [15]. The concentration of the solutions of chemically synthesized peptides was calculated on the basis of the weight of the dry peptide powder.

**Evaluation of the antimicrobial activity of the peptides**

The antimicrobial activity of mini-bactenecins was characterized using two methods: radial diffusion in agarose gel and the broth microdilution method. Microorganism strains were provided courtesy of R. Lehrer (University of California, Los Angeles, USA), A. Tossi (University of Trieste, Italy), E.I. Ermolenko (Institute of Experimental Medicine); members of the Military Medical Academy; G.E. Afinogenov (Vreden Russian Research Institute of Traumatology and Orthopedics, Ministry of Health of the Russian Federation). We used a clinical isolate of *Pseudomonas aeruginosa *resistant to aztreonam, ceftazidime, cefotaxime, a clinical isolate of *Klebsiella spp. *resistant to tetracycline (both strains were obtained from the urine of the patient with cystitis), a clinical isolate of *Acinetobacter baumannii *resistant to meropenem (from an infected wound); a clinical isolate of *Staphylococcus intermedius *(from an infected wound caused by a dog bite) resistant to ciprofloxacin, cefuroxime, clindamycin, erythromycin, rifampin, gentamicin, benzilpenicilin, oxacillin; and a clinical isolate of a yeast-like fungus *Candida parapsilosis *resistant to amphotericin B and clotrimazole (scraping from the nail plate).

*The method of radial diffusion in agarose gels*. We used the methodology proposed by Lehrer et *al*. [16] and described in detail in [12]. The antibiotic activity of AMPs was quantified by measuring the diameter of the microbial growth inhibition zone around the wells punched in the agarose gel, where the peptides had been applied. The measured values were expressed in units (1 U = 0.1 mm) after subtracting the well diameter (2 mm = 20 U). The minimal inhibitory concentration (MIC) of the AMP was determined by plotting data of the peptides antimicrobial activity vs their concentration using the Sigma Plot 11 software (Systat Software Inc., USA) and calculating the x intercept value of the linear regression plot (peptide concentration in μM), which was taken as the MIC value. Two parallel samples were used in each experiment. The experiments were conducted in triplicate, and the average value of the MIC ± standard deviation was calculated.

*The broth microdilution assay. *We applied a standard method used in microbiology to test antibiotics, which was slightly modified taking into account the specificity of AMPs [17] according to [12]. The lowest peptide concentration which completely inhibited visible growth of microorganisms in the wells of 96-well plates was taken as MIC. Three parallel samples were tested in each experiment. The results are reported as medians obtained in three to five independent experiments.

**Assessment of the effect of peptides on the permeability of the outer and cytoplasmic membranes of *E. coli *ML35p for chromogenic markers**

The effect of peptides on the barrier function of membranes of Gram-negative bacteria was studied using the method [18] as revised in [19]. The ML35p strain of *E. coli *is characterized by a lack of lactose permease, constitutive β-galactosidase synthesis in the cytoplasm, and it also contains β-lactamase in the periplasmic space. The state of the outer and cytoplasmic membranes of *E. coli ML35p *cells was assessed based on their permeability to chromogenic markers, nitrocefin, and *o-nitrophenyl-β-D*-galactopyranoside (ONPG),* β*-lactamase and *β*-galactosidase substrates, respectively. Samples were placed in the wells of a 96-well plate according to [12], and the optical density *(OD)* of the solution rising due to the appearance of products of nitrocefin or ONPG hydrolysis was measured at λ = 486 and 420 nm, respectively, using a Spectra- Max 250 spectrophotometer (Molecular Devices, USA) at 37°C with regular shaking of the plates for 2 h. The data were processed using the Sigma Plot 11 software.

**Estimation of the lipopolysaccharidebinding activity of the peptides**

The lipopolysaccharide-binding (lipopolysaccharide- neutralizing) activity of the peptides was studied using the quantitative chromogenic Limulus Amebocyte Lysate test (Lonza Walkersvile, USA). The approaches described by Zhao *et al*. [20] were used to conduct the experiments and analyze the results. The peptides were serially diluted in endotoxin-free acidified water (0.01% acetic acid) and incubated with *E. coli* O111: B4 lipopolysaccharide (LPS) at a final concentration of 0.5 U/ml for 30 min at 37°C in Costar 3596 plates (Corning, USA). We assayed free LPS according to the kit manufacturer’s recommendations. The plate was placed in the thermostatic chamber of a SpectraMax 250 spectrophotometer (Molecular Devices, USA) and incubated at 37°C while measuring *OD *of the solution at 405 nm; the difference between the OD values at the beginning of incubation and after 10 min, Δ OD405, was calculated.

The proportion of bound LPS (%) was determined using the formula

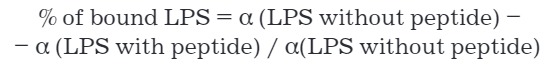



where *α *= ΔOD_405_ (peptide (or water) with LPS*) -* ΔOD_405_ (peptide (or water) without LPS). We constructed the curves representing the relationship between the proportion of bound LPS and the AMP concentration in the incubation medium (Sigma Plot program 11, Systat Software Inc., USA) and determined EC_50_ (50% effective concentration or peptide concentration corresponding to 50% binding of the LPS).

**Analysis of the peptides, hemolytic activity**

Red blood cells were isolated from the blood of healthy donors by the standard methods. A red cell pellet was diluted (we assumed that the pellet contained 100% cell suspension) to obtain a 2.8% erythrocyte suspension in phosphate buffered saline (PBS). We placed 27 μl of the erythrocyte suspension and 3 μl of the test peptide (at different concentrations) in PBS or 3 μl of PBS without the peptides (control) to each analyzed sample. The samples (in triplicates) were incubated at 37°C for 30 min, 75 μl of ice cold PBS was added, and then the samples were centrifuged at 5,000 *g *for 4 minutes. Absorbance of the supernatants was measured at λ = 540 nm.

**Assessment of the effect of peptides on the viability of cultured cells**

The viability of cultured human cells after 20-hour incubation with the peptides was evaluated using the standard MTT assay [21] according to [12]. Cell culturing and separation of neutrophils and mononuclear cells of peripheral blood from healthy donors was carried out using standard methods.

**Mass Spectrometry**

The molecular masses of the isolated peptides were determined on the MALDI-TOF mass spectrometer Reflect III (Bruker, Germany) equipped with a UV-laser with a wavelength of 336 nm. We used 2,5-dihydroxybenzoic acid (Sigma, Germany) in 20% acetonitrile, 0.1% TFA at a concentration of 10 mg/ml as a matrix. Average molecular masses are shown.

**Sequencing**

The amino acid sequence was determined using the Procise cLC 491 protein sequencing system (Applied Biosystems, USA). Phenylthiohydantoic derivatives of the amino acid residues were identified on a 120A PTH analyzer (Applied Biosystems, USA).

**Synthesis of mini-bactenecins**

Mini-ChBac7.5Nα and mini-ChBac7.5Nβ were synthesized using solid phase synthesis and the Fmoc/ tBu-strategy on a Syro2000 peptide synthesizer (MultiSynTech GmbH, Germany) [22]. After completion of the synthesis, the peptides were removed using a mixture containing 5% water, 4% of m-cresol, 5% of thioanisole, and 2% of ethanedithiol in TFA at room temperature for 4 hours, cooled and precipitated with diethyl ether. Synthesized peptides were purified on Akta HPLC (Amersham Bioscience GmbH, Germany) using Jupiter C18 column (20 mm × 250 mm, Phenomenex Inc., USA) with a linear gradient of acetonitrile with 0.1% TFA. The molecular masses of the peptides were confirmed using MALDI-TOF-MS, and purity was confirmed using RP HPLC

**Statistical analysis**

When determining AMP cytotoxic activity for human cells, the statistical significance of the differences between the experimental and control groups was evaluated according to the Student’s *t-test (p * < 0.05), n = 6 using the Prism 5 software (GraphPad software Inc., USA).

## RESULTS

**Isolation and purification of new antimicrobial peptides from caprine leukocytes**

**Fig. 1:**
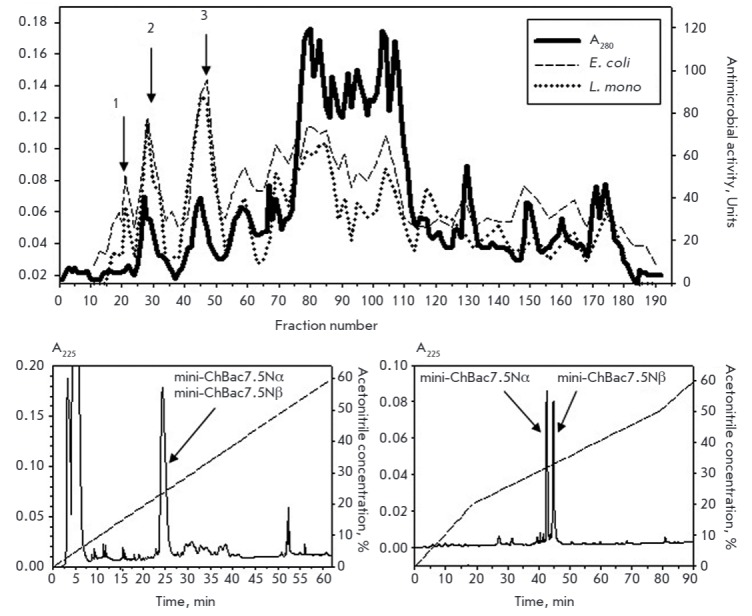
Purification of antimicrobial peptides from extracts of goat leukocytes. A – Preparative continuous elution electrophoresis (CEE) of YM10 ultrafiltrate of goat leukocyte extract in a polyacrylamide gel (current strength 30 mA, flow rate 36 ml/h, fraction volume 3 ml). Peak 1 – fractions of peptides with a molecular mass of 2.8 – 6 kDa, containing mini-bactenecins; peak 2 – ChBac3.4; peak 3 – ChBac5. CEE fractions were tested for antimicrobial activity against* Listeria monocytogenes *EGD and *E.coli *ML35p in radial diffusion assays (right X axis – antimicrobial activity units). B – RP-HPLC of CEE fractions 19–24, using a linear gradient of acetonitrile (0–60%; 1%/min; 0.1% trifluoroacetic acid) on the Vydac C18-column (0.46 x 25 cm). C – RP-HPLC of fractions 24–26 obtained after RP-HPLC is shown on panel B (acetonotrile gradient: 0–20% during 20 min, 20–50% during 60 min, 50–60% during 10 min, 0.13 % heptafluorobutyric acid). Peaks of peptides with average molecular masses of 2895.5 Da and 2739.3 Da designated as mini-ChBac7.5Nα and mini-ChBac7.5Nβ are shown by arrows.

We isolated the peptides under conditions that enabled the processing of cathelicidin precursors, resulting in the release of mature AMPs. Preparative continuous elution electrophoresis (CEE) was used to separate cationic peptides obtained after the ultrafiltration of caprine leukocyte extracts through the YM-10 membrane. Fractions were analyzed by measuring solution absorbance at 280 nm and evaluating antimicrobial activity by the radial diffusion method (*Fig. 1A*). The fractions 17–24 contained components with the highest electrophoretic mobility toward the cathode, peptides with a molecular weight ranging from 2.8 to 6 kDa, and possessing antimicrobial activity (peak 1). Peaks 2 and 3 comprised the bactenecins ChBac3.4 and ChBac5 (*Fig. 1A*).

Successive RP-HPLC cycles using various counterions were employed to obtain individual peptides eluting in fractions, corresponding to peak 1. *Fig. 1B* shows the results obtained during the first step of chromatographic separation of the peptides contained in the pooled fractions 19–24. Antimicrobial activity was found in the fractions shown by arrows (24–26th minutes) and containing two peptides with average molecular masses of 2895.5 and 2739.3 Da. The peptides were separated by re-chromatography using heptafluorobutyric acid as a counterion (*Fig. 1C*). We obtained individual peptides (and denominated them mini-bactenecins) eluating from the column in fractions corresponding to the peaks shown by arrows on the chromatogram: peptides with average molecular masses of 2895.5 Da, mini-ChBac7.5Nα, and 2739.3 Da, mini-ChBac7.5Nβ.

**Fig. 2:**
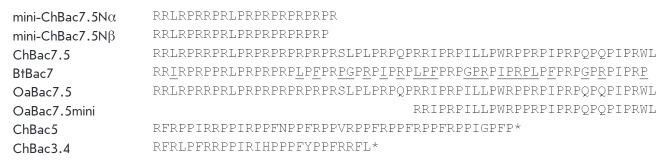
Amino acid sequences of the antimicrobial peptides isolated from goat leukocytes, mini-ChBac7.5Nα and ChBac7.5Nα-β, compared to the previously reported sequences of bactenecins: bovine Bac7 (BtBac7; diverse amino acid residues are underlined) [4], ovine OaBac7.5 [7] and OaBac7.5mini [2], caprine ChBac5 [11] and ChBac3.4 [12]. The structure of the full-size caprine ChBac7.5 is shown. * – amidated C-terminus of the molecule.

The analysis of the primary structure of the isolated AMPs showed that both peptides are N-terminal fragments of caprine bactenecin 7.5. Information about the structure of the latter was previously obtained by gene cloning and represented in the database (Q9XSQ9, (Q9XSQ9_CAPHI) UniProtKB /23], but the corresponding protein has not been isolated from leukocytes (*Fig. 2*). Isolation of the fragment of ovine bactenecin 7.5 (a peptide structurally similar to caprine bactenecin 7.5) was described: however, this molecule comprised the C-terminal portion of bactenecin 7.5 [2]. Given that this peptide was designated as OaBac7.5mini, we similarly named our peptides mini-ChBac7.5Nα and β. The additional letter N indicates that these are N-terminal fragments (Ch stands for the abbreviation *C. hircus,* domestic goat).

The procedure of isolation and purification was repeated in several series of experiments, resulting in the same fractions of mini-bactenecins. The abovementioned data were obtained using material where proteins were extracted with 10% acetic acid. Mini-bactenecins were also detected when extracting proteins with a detergent: cetyltrimethylammonium bromide. Protease inhibitors were not used, since mature forms of cathelicidins-derived AMPs could not be obtained in this case.

Thus, new peptides, N-terminal fragments of bactenecin 7.5, were for the first time isolated from goat leukocytes. We have not detected the full-length bactenecin 7.5. Probably, it mostly succumbed to proteolytic cleavage.

**Antimicrobial activity of mini-bactenecins**

The antimicrobial activity of mini-bactenecins obtained by chemical synthesis was analyzed using two methods: radial diffusion in agarose gel (RD) and the broth microdilution assay (*Table*). When assessing the activity of bactenecins by RD, the peptides were incubated with microorganisms under different conditions: in a medium with low ionic strength (0.01 M sodium phosphate buffer, pH 7.4, without other salts added) and in the same medium but supplemented with 100 mM sodium chloride.

**Table:** Antimicrobial activity of caprine mini-bactenecins: minimal inhibitory concentrations (MIC, μM) obtained by two methods

	mini-ChBac7.5Nα			mini-ChBac7.5Nβ		
	Radial diffusion assay in agarose gel MIC (μM)*		Broth microdilution assay MIC (μM)**	Radial diffusion assay in agarose gel MIC (μM)*		Broth microdilution assay MIC (μM)**
	without NaCl	100 mM NaCl	Broth***	without NaCl	100 mM NaCl	Broth***
E.coli ML35p	0.3 ± 0.1	1.5 ± 0.2	1	0.3 ± 0.1	1.4 ± 0.2	1
E.coli ATCC 25922	0.6 ± 0.1	0.9 ± 0.2	2	0.5 ± 0.2	0.8 ± 0.2	2
E.coli M17	0.5 ± 0.1	0.8 ± 0.1	2	0.5 ± 0.1	0.9 ± 0.2	1
Pseudomonas aeruginosa ATCC 27853	1.1 ± 0.4	3.7 ± 1.2	2	1.0 ± 0.3	3.2 ± 0.8	2
Pseudomonas aeruginosa clinical isolate	ND	ND	2	ND	ND	2
Klebsiella spp. clinical isolate	ND	ND	4	ND	ND	4
Acinetobacter baumannii clinical isolate	ND	ND	2	ND	ND	4
Listeria monocytogenes EGD	0.2 ± 0.1	1.0 ± 0.2	2	0.2 ± 0.1	0.9 ± 0.2	2
Micrococcus luteus CIP A270	ND	ND	1	ND	ND	1
Staphylococcus aureus 710A	0.7 ± 0.2	> 50	> 64	0.6 ± 0.1	> 50	> 64
Staphylococcus aureus ATCC 25923	ND	ND	> 64	ND	ND	> 64
MRSA ATCC 33591	0.7 ± 0.2	> 50	> 64	0.5 ± 0.1	> 50	> 64
Staphylococcus intermedius clin. isolate	ND	ND	> 64	ND	ND	> 64
Candida albicans 820	0.3 ± 0.1	> 50	64	0.3 ± 0.1	> 50	> 64
Candida parapsilosis clinical isolate	ND	ND	> 64	ND	ND	> 64

^*^data are shown as mean values ± S.D. (n = 6). Radial diffusion assay was performed under the following conditions: low salt (10 mM phosphate buffer, pH 7.4) and high salt (10 mM phosphate buffer + 100 mM NaCl, pH 7.4).

^**^data are shown as medians derived from 3–5 experiments performed in triplicates.

^***^Mueller-Hinton broth for bacteria or Sabouraud broth for fungi.

ND – not determined.

It was reported that the currently known proline- rich AMPs (PR-AMPs) have high antimicrobial activity against Gram-negative bacteria and decreased activity against most Gram-positive bacteria, particularly staphylococci [24]. We have shown that, in a medium with a low ionic strength, mini-bactenecins demonstrate a broad spectrum of antimicrobial activity and high activity against both Gram-negative and Gram-positive bacteria, including staphylococci, and against fungus *C. albicans (Table)*. However, an increase in the medium ionic strength results in reduced AMP activity against both staphylococci and *C. albicans.* In the case of Gram-negative bacteria, the relationship between the activity of mini-bactenecins and the ionic strength of the medium is less pronounced.

The study of the antimicrobial action of the peptides in broth microdilution assay (*Table*) revealed a high activity of mini-bactenecins against Gram-negative bacteria, including strains resistant to some antibiotics used in clinical practice: *P. aeruginosa *(resistant to aztreonam, ceftazidime, cefotaxime), *Klebsiella spp.* (resistant to tetracycline*), A. baumannii *(resistant to meropenem); MIC 2–4 μM. The peptides demonstrated pronounced activity against Gram-positive bacteria* Listeria monocytogenes *and *Micrococcus luteus*, but their antimicrobial activity against staphylococci and fungi from the genus *Candida *was negligible at concentrations ranging from 1 to 64 μM.

**The effect of AMPs on the permeability of the outer and cytoplasmic membranes of *E. coli* ML35p for chromogenic markers**

**Fig. 3:**
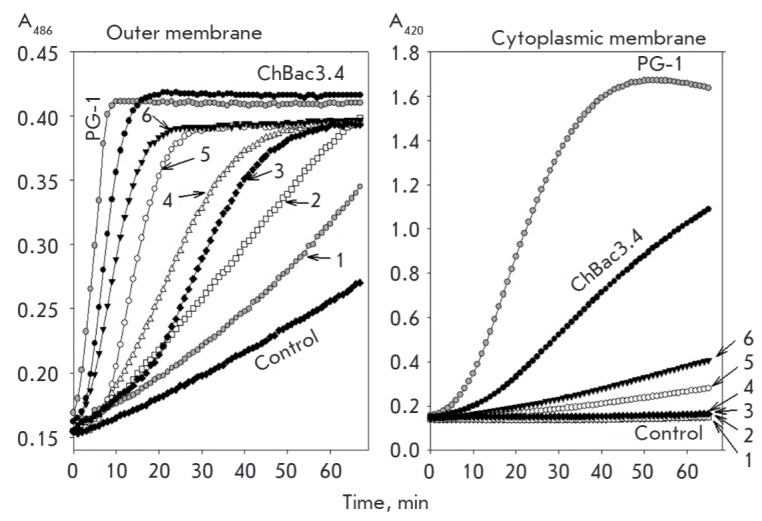
Kinetics of changes in *Escherichia coli *ML-35p membrane permeability with respect to chromogenic markers resulting from incubation of bacteria with mini-ChBac7.5Nα taken in various concentrations: 1 – 0.6 μM; 2 – 1.2 μM; 3 – 2.5 μM; 4 – 5 μM; 5 – 10 μM; 6 – 20 μM. x-axis – incubation time, min. y-axis – optical density of the solution containing chromogenic markers: nitrocefin hydrolysis product at a wavelength of 486 nm (left panel displaying the outer membrane permeabilization) and ONPG hydrolysis product, o-nitrophenol, at 420 nm (right panel displaying the inner membrane permeabilization). Another caprine bactenecin, ChBac3.4, was used as a reference (at a final concentration of 5 μM, which is 2 x MIC); a membranolytic peptide, porcine protegrin 1 (PG-1), was used as a positive control (2.5 μM).

One of the most important objectives in studying the functional properties of AMPs is to identify the main target of their antimicrobial action. Bacterial membranes are targets for most AMPs. Peptides cause their rapid and irreversible disintegration. However, some AMPs, including PR-AMPs, mostly alter intracellular processes in bacterial cells and damage their membranes only at concentrations highly exceeding MIC [25]. We studied the effect of mini-bactenecins on the permeability of the outer and cytoplasmic membranes of *E. coli* ML35p. *Fig. 3* shows the kinetics of the action of mini-ChBac7.5Nα at concentrations of .6-20 μM on the membranes of *E. coli *ML35p*. *Caprine bactenecin ChBac3.4 (5 μM, which is 2 times higher than MIC) was used as a reference peptide, and the porcine membrane- active peptide protegrin 1 (PG-1) was used as a positive control. The action of mini-bactenecin results in increased permeability of the bacterial outer membrane to the chromogenic marker almost over the entire investigated concentration range, although in the case of PG-1 (2.5 μM, which is 2 times higher than MIC) this effect is more pronounced. However, the studied peptide from caprine leukocytes has no significant impact on the permeability of the cytoplasmic membrane of *E. coli* to marker molecules. Only at high peptide concentrations (10 and 20 μM), which are significantly higher than MIC (1-2 μM), the results slightly differ from the control values without AMPs. Unlike mini-bactenecin, the effect of ChBac3.4 occurs at a concentration which is only twofold higher than MIC. In the case of the second mini-bactenecin, mini- ChBac7.5Nβ, the results were almost identical for mini-ChBac7.5Nα (data not shown). These findings suggest that bacterial membranes are not the main target of the mini-bactenecins under study, as well as other known PR-AMPs. It is likely that they can bind to the DnaK chaperone, similarly to the bovine Bac7 and ovine OaBac7.5 fragments, and modulate its ATPase activity, disturbing the protein folding process in the cell [25, 26], or interact with the 70S ribosome, impairing the translation process, as shown for apidaecins, oncocins, and the bovine Bac7 fragment 1–35 [27, 28]. Just like the fragment 1–35 of bovine Bac7, which affected the cytoplasmic membrane of *E. coli *ML35p at concentrations several times higher than MIC [24], mini-bactenecins affect the permeability of the inner membrane of this bacterium only at concentrations 10- to 20-fold higher than MIC.

**The lipopolysaccharide-binding activity of caprine mini-bactenecins**

**Fig. 4:**
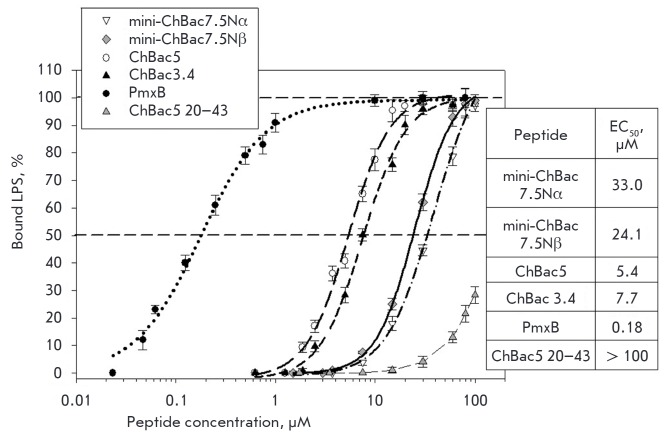
lipopolysaccharide by mini- ChBac7.5Nα and mini-ChBac7.5Nβ as compared with caprine bactenecins ChBac3.4, ChBac5 and its inactive fragment ChBac5 (20- 43) in a quantitative chromogenic *Limulus Amebocyte Lysate* Assay. Polymyxin B (PmxB) was used as a positive control. Mean values ± S.D., n=6 are shown. EC_50_ (i.e., peptide concentrations that bound 50% of the LPS) are shown in the inset.

Binding to lipopolysaccharide (LPS), the component of the outer membrane of Gram-negative bacteria, is one of the essential properties of AMPs, because the capacity of that binding largely determines the subsequent effectiveness of the antimicrobial action of peptides. In the development of pharmaceuticals based on AMPs, special attention is focused not only on antimicrobial properties, but also on the LPS-binding (neutralizing) activity, taking into account the need to obtain a compound which could both contribute to the inactivation of pathogenic microorganisms and prevent or eliminate the consequences of septic shock caused by Gram-negative bacteria, a serious complication of infectious diseases, often with a lethal outcome. Numerous recent publications provide a comprehensive analysis of the relationship between the structural features of the peptides that are used as drug prototypes and their antimicrobial action, selectivity with respect to prokaryotic cells, and LPS-neutralizing properties. It has been shown that the LPS-neutralizing activity of a peptide depends on the hydrophobicity/net positive charge ratio of its molecule [29]. We measured the LPS-binding activity of mini-bactenecins by determining the effective concentration when 50% of LPS (LPS of *E. coli *O111:B4*) *is bound to the peptide [20]. As a reference, we provide the results obtained for other AMPs from goat leukocytes, namely bactenecins ChBac3.4, ChBac5, and the peptide with low antimicrobial activity, the chemically synthesized C-terminal region (residues 20–43) of ChBac5 bactenecin (ChBac5 20-43). Polymyxin B, known as a compound with high affinity to LPSs, was used as a positive control (*Fig. 4*). Mini- bactenecins are characterized by significantly higher values of this activity compared to ChBac5 20–43, although they are somewhat inferior to the bactenecins ChBas3.4 and ChBac5, which can be explained by the higher net positive charge and lower hydrophobicity of mini-bactenecin molecules compared to ChBas3.4 and ChBac5 (*Fig. 4*). Mini-ChBac7.5Nα contains 12 arginine residues and only two leucine residues (mini- ChBac7.5Nβ contains 11 arginine residues and 2 leucine residues). Furthermore, mini-bactenecins do not contain aromatic amino acid residues, which (in particular tryptophan residues) are believed to enhance LPS-neutralizing activity [30]. On the contrary, ChBac3.4 and ChBac5 contain a relatively large amount of aromatic amino acid residues, mainly phenylalanine. These data provide valuable information for analyzing the patterns of the various types of biological activity of AMPs and point to the possibility of a development of antibiotic drugs based on mini-bactenecins by designing their analogues containing a larger number of hydrophobic amino acid residues, in particular tryptophan.

**The action of mini-bactenecins in mammalian cells**

It is known that most PR-AMPs have no pronounced toxicity with respect to mammalian cells [25]. Evaluation of the hemolytic activity of mini-bactenecins toward human erythrocytes shows that, at concentrations of 1–100 μM, both peptides have no pronounced effect on red blood cells. The values of samples containing specified concentrations of the peptides did not differ significantly from those of the control samples containing no AMPs (p > 0.05, Student’s t-test, n = 9).

We assessed the effect of mini-bactenecins at a concentration of 1–30 μM on human cells using the MTT assay. It was found that the peptides have low cytotoxic activity against various types of cultured human cells: namely, erythroleukemia K-562 cells, histiocytic lymphoma U-937 cells, promyelocytic leukemia HL- 60, epithelioid lung carcinoma A-549, epidermoid carcinoma A-431, human osteosarcoma MG-63, as well as normal human skin fibroblasts, human embryonic lung fibroblasts MRC-5, and neutrophils and mononuclear cells of human peripheral blood. The cytotoxicity values obtained after 24 h of incubation with peptides were not significantly different from the values calculated for the control samples containing no peptide over the entire range of concentrations: 1–30 μM (p > 0.05, Student’s t-test, n = 9). These data are indicative of the fact that the action of caprine mini-bactenecins is selective with respect to microbial cells, which is consistent with observations showing a low toxicity of N-terminal fragments 1–16, 1–23, 1–35 of bovine Bac7 toward mammalian cells [24].

## DISCUSSION

We isolated two antimicrobial peptides, the mini-bactenecins mini-ChBac7.5Nα and mini-ChBac7.5Nβ, from leukocytes of the domestic goat *C. hircus*. They are N-terminal fragments of the ChBac7.5 peptide, which were for the first time obtained from blood cells by us. Several fragments of OaBac11, OaBac5, and OaBac7.5 bactenecins had been previously isolated from ovine leukocytes [2]. The C-terminal fragment (32–60) of OaBac7.5, isolated by Anderson *et al*. [2] and designated as OaBac7.5mini, showed relatively low antibacterial activity [31] compared to the activity of the N-terminal fragments of goat bactenecin 7.5. Caprine mini-bactenecins are structurally similar to the N-terminal part of bovine Bac7 [5] (*Fig. 2*). An N-terminal region of the bovine Bac7 molecule (at least 16 amino acid residues) [24], whose length approximately corresponds to the peptides isolated by our group, is required for any antimicrobial activity by this bactenecin. The C-terminal fragments of bovine Bac7 had a low antimicrobial activity [24]. The N-terminal sequences of the molecules of caprine and ovine bactenecin 7.5, as well as bovine bactenecin 7, are structurally similar, whereas the C-terminal regions are substantially different. Discovery of the fragments of ovine bactenecins 7.5 [2] and caprine mini-bactenecins suggests that the peptides, formed after the fragmentation of the parent bactenecin molecules, perform the main protective functions: N-terminal derivatives execute an antimicrobial action, while C-terminal fragments may play a different role which remains unclear.

The importance of a fragmentation of mature AMP forms, including regulation of their biological effects in the course of an infectious process, was assumed when studying the proteolytic cleavage of human cathelicidin LL-37. Cleavage of this peptide results in the formation of fragments, some of which have a higher antimicrobial activity than full-length LL-37 [1, 32]. However, it was found that, along with potent antimicrobial effects, the immunomodulatory activity of these peptides is reduced compared to the full-length cathelicidin [32]. The pattern of cathelicidin fragmentation depends on many factors, but mostly on the activity of the proteases involved in its processing and on the activity of their inhibitors [1]. These factors, in turn, depend on the parameters determined by the microenvironment, which can vary during infectious or other pathological processes. Therefore, the fragmentation of human cathelicidin may be considered as one of the mechanisms of precise and multifaceted regulation of the functional activity of AMPs. On the other hand, investigation of the biological activity of peptide fragments informs the development of various antibacterials, as well as antitumor peptide pharmaceuticals, LL-37 derivatives, which are regarded as promising templates for new drugs.

Other antimicrobial polypeptides are also subjected to fragmentation. Their cleavage produces truncated forms having a pronounced bactericidal activity. For example, processing of lactoferrin, a component of specific neutrophilic granules, generates the antimicrobial peptide lactoferricin, which is considered as a compound that plays an independent role in the biological defensive functions of neutrophils [33]. Fragments of histones that have antimicrobial activity and are expected to provide a protective effect were isolated from the leukocytes and skin of some fish and amphibians [34, 35].

The enzymes that can perform the corresponding processing of PR-AMPs, in particular caprine bactenecin 7.5, are of great interest. It can be assumed that this process involves several different proteases and that cleavage may consist of several stages. In the case of mini-ChBac7.5Nβ, prolyl endopeptidase (PREP [EC 3.4.21.26]) or prolyl carboxypeptidase (PRCP [EC 3.4.16.2]) could be one of these enzymes. They cleave the peptide bond between the arginine and proline residues (in the ChBac7.5 molecule presumably between the proline 21 and arginine 22 residues). These proteases are present in neutrophilic granulocytes and have been shown to play an important role in inflammatory responses [36]. Further investigation using different types of protease inhibitors will shed light on this issue.

## CONCLUSION

Isolation of highly active antimicrobial peptides comprising N-terminal fragments of bactenecin 7.5 (we call them mini-bactenecins: mini-ChBac7.5Nα and mini- ChBac7.5Nβ) from the leukocytes of domestic goat supports the idea that fragmentation of antimicrobial peptides of the innate immune system is an important requirement for the triggering and regulation of protective responses in the course of inflammatory or infectious processes. We have shown that mini-bactenecins exert a potent antimicrobial activity against Gram-negative bacteria, including antibiotic-resistant strains, posses lipopolysaccharide-binding activity, and are non-toxic toward cultured human cells. The obtained data point to the prospectivity of further investigations of the antimicrobial activity of these compounds on a wider spectrum of microorganisms in order to prove the possibility of developing new antibacterial pharmaceuticals on their basis.

## Abbreviations

AMP: antimicrobial peptide
CEE: continuous elution electrophoresis
CFU: colony forming units
MIC: minimal inhibitory concentration
PBS: phosphate buffered saline
MALDI: TOF MS matrix assisted laser desorbtion/ionization-time of flight mass-spectrometry
MRSA: methicillin resistant Staphylococcus aureus
ONPG: ortho-nitrophenyl β-D-galactopyranoside
PG-1: protegrin 1
PR-AMP: proline-rich antimicrobial peptide
RP-HPLC: reverse-phase high performance liquid chromatography
